# The use of non-prescribed anti-malarial drugs for the treatment of malaria in the Bolgatanga municipality, northern Ghana

**DOI:** 10.1186/1475-2875-12-266

**Published:** 2013-07-31

**Authors:** Samuel Aborah, Patricia Akweongo, Martin Adjuik, Roger A Atinga, Paul Welaga, Philip B Adongo

**Affiliations:** 1 Ghana Health Service, Bolgatanga Regional Hospital, Bolgatanga, Ghana; 2 Department of Epidemiology and Disease Control, School of Public Health, University of Ghana, Accra, Ghana; 3 INDEPTH NETWORK Secretariat, Accra, Ghana; 4 Department of Public Administration and Health Services Management, University of Ghana Business School, Accra, Ghana; 5 Navrongo Health Research Centre, Navrongo, Ghana; 6 Department of Social and Behavioral Sciences, School of Public Health University of Ghana, Accra, Ghana

**Keywords:** Malaria, ACT, Non-prescribed drugs, Ghana

## Abstract

**Background:**

The use of non-prescribed anti-malarial drugs can lead to treatment failure and development of drug-resistant parasites. This study investigated the use of non-prescribed anti-malarial drugs for the treatment of malaria in the Bolgatanga Municipality of northern Ghana.

**Methods:**

This was a cross-sectional survey of a random sample of 392 adults and children with episodes of malaria in the last four weeks prior to the study.

**Results:**

Majority of survey respondents 96.9% (380) knew the symptoms of malaria, 75% (294) knew the causes of malaria and 93.1% (365) were aware of mode of transmission of malaria. The use of non-prescribed anti-malarial drugs was 16.8% (95% CI: 13.3-21.0) among the respondents. About 56% (95% CI: 43.3-68.3) of the respondents who took non-prescribed anti-malaria drugs took non-artemisinin-based combination therapy (chloroquine, artemether, amodiaquine and sulphadoxine-pyrimethamine). Respondents above five years of age were more likely to use non-prescribed anti-malarial drugs than those below five years of age [P < 0.001]; respondents who knew the right source of malaria treatment were less likely to use non-prescribed anti-malarial drugs than those who did not [P = 0.002]. Respondents using non-prescribed anti-malarials were influenced by people around them who used non-prescribed anti-malarials. Thus, these respondents were more likely to use non-prescribed anti-malarials than those who were not influenced [P = 0.004].

**Conclusions:**

Respondents’ knowledge of malaria treatment and the influence of people using non-prescribed anti-malarials are factors affecting use of non-prescribed anti-malarials. The study concludes that there is high use of non-prescribed anti-malarial drugs in the municipality and most of the non-prescribed anti-malarias were non-artemisinin-based combination therapy. The study recommends education of the general public and chemical sellers to reduce the use of non-prescribe anti-malaria drugs.

## Background

Malaria is a major cause of morbidity and mortality worldwide with an estimated 219 million cases of malaria and 660,000 deaths occurring in 2010. About 85% of these cases occurred in sub-Saharan Africa. Malaria remains a threat to more than 40% of the world’s population, particularly pregnant women and children under five years of age [1].

In recent times, emphasis has been put on early treatment with effective anti-malaria drugs at community level within 24 hours of the onset of symptoms. As a result, in many communities efforts have been made to train caregivers to initially provide anti-malarial drugs to treat perceived malarial illness [2,3].

Studies have shown that non-prescribed anti-malaria drugs used by the majority of caregivers include chloroquine, amodiaquine, sulphadoxine-pyrimethamine and artemisinin-based combination therapy (ACT) and the dosage regimen taken are incorrect in most cases [4,5].

The reasons for using non-prescribed anti-malaria drugs include lack of access to health facilities, cost of treatment in health facilities and social distance of health workers from patients [6]. Several sociocultural factors, including people’s beliefs, perceptions and knowledge about malaria and influence of peers on people’s choice of malaria treatment [4,7] have been cited to influence use of non-prescribed anti-malarials. Drug sellers lacking adequate knowledge about malaria treatment contribute to use of non-prescribed anti-malarials. The inappropriate use of anti-malarial drugs affected by these factors over the years led to treatment failure and drug resistant parasites which, necessitated the global change in malaria drug policy [8,9]. Although, the use of rapid diagnostic tests to confirm malaria before treatment has the potential to improve treatment, these are not available to both providers [10].

In view of this, almost all malaria-endemic countries have changed their national drug policies. In compliance with WHO recommendations, countries have switched from single drug treatment, as it becomes ineffective, and now promote ACT, as first-line treatment for uncomplicated malaria since 2001 [11]. Combining anti-malarial drugs with different modes of action improves the efficacy of individual drugs, increases cure rates and prolongs the spread of drug resistance [12-14]. Based on this evidence, Ghana changed its national malaria policy from chloroquine to ACT, amodiaquine/artesunate and artemether-lumefantrine in 2005. The present study therefore sought to investigate the extent to which non-prescribed anti-malarial drugs are used and the factors influencing use of non-prescribed anti-malarials in order to understand the effective knowledge-based intervention that can be used to counteract the practice. Non-prescribed anti-malarial drug” refers to anti-malarial drug that is not prescribed by a qualified health professional in a health facility or community heath volunteer in the community under the home management of malaria strategy. It includes anti-malarial drugs obtained from chemical or pharmacy shops, drug peddlers, relatives or friends without prior consultation with a qualified health professional or community health volunteer. It also includes “left over anti-malaria drugs” which was obtained from health facility in a previous visit).

## Methods

### Study area

The study was conducted in Bolgatanga Municipality of the Upper East Region in northern Ghana with a total land area of 729 sq km. The climate is tropical, with two distinct seasons, the rainy season that runs from May to October and a long dry season from November to April, with hardly any rains. Mean annual rainfall is 950 mm, maximum temperature is 45^0^C in March and April with a minimum of 12^0^C in December. The population of the municipality is 152,658 with a growth rate of 1.7%. The population density is 141.2 persons per sq km, one of the highest in the country. The majority of inhabitants of Bolgatanga municipality are from Guruni ethnic origins.

Generally, housing conditions are poor. Several sections of downtown Bolgatanga have developed into slums (Zongos) where the most vulnerable people live. These Zongos lack drainage systems and toilet facilities, with implications for mosquito breeding and malaria transmission.

Bolgatanga Municipality has two hospitals, six health centres, six clinics and 14 Community Health Planning Services (CHPS) compounds. The latter’s facilities provide community treatment services. There are mobile clinics ran by the Catholic and Presbyterian Churches. The municipality has several pharmacies and licensed chemical sellers.

### Study design

This was a cross-sectional survey of 392 adults and children with malaria in the last four weeks prior to the study. The method of data collection was mainly Quantitative in nature. Data collected included knowledge about malaria- the causes, symptoms, treatment and prevention; experience with last episode of malaria- where care was sought, type of medication taken, where medication was obtained from, whether medication taken within 24 hours, perceived severity of symptoms and influence of peers/friends/relatives on treatment. All the outcome variables were transformed into binary variables measure study outcomes. Source of treatment was assessed by respondents self-reporting on where they obtained drugs to treat the last episode of malaria. The outcome variable takes a value of 1 if the respondents obtained their drugs from health workers and 0 if otherwise. The independent variables were treated likewise.

A three-stage sampling technique was used to randomly select respondents for the survey. Bolgatanga municipality has nine sub-districts made up of five rural and four urban sub-districts. In the first stage the municipality was divided into two strata, rural and urban, and three sub-districts were randomly selected from each of the rural and urban stratum. The second stage involved the random selection of 40 communities, (20 from the rural and 20 from the urban). Then, ten households were randomly selected in each community by the interviewer going to the centre of the community and spinning a pen. The direction in which the pen pointed was followed and from which the first house and the eligible household was selected. Only one person who had had malaria in the last four weeks was interviewed. The interviewer then spins the pen again from the last selected house to the nearest house/household. This was continued till the maximum number of ten respondents was obtained in each community. After seeking and receiving consent from the respondent the interview was conducted. The procedure was used to select all eligible adults and children for interview. For children who were below 18 years, the parent or primary caregiver was interviewed.

### Analysis of data

The data were analysed using Epi info software version 3.4.1. Descriptive statistics such as mean, median, minimum and maximum were calculated for continuous variables and percentages for categorical variables. The association between the use of non-prescribed anti-malarial drugs and other variables was established using Chi-square test. Multivariable logistic regression model was used to determine predictors of non-prescribed anti-malarial drug use among respondents.

### Ethical considerations

Ethical clearance was sought from the Ghana Health Service Ethical Review Board before the study was done. Study objectives, benefits, risks and the right to refuse participation and confidentiality of responses were explained to participants. Written informed consent was obtained from each participant.

## Results

The majority of respondents knew the symptoms of malaria 96.9% (380), the cause of malaria 75% (294) and mode of transmission of malaria 93.1% (365). Many respondents 75.8% (297) also reported cleaning the surroundings and sleeping under a mosquito net 95.9% (375) as ways to prevent malaria. Similarly many 84.9% (333) respondents knew the best place (health facility consultation) to seek anti-malarial treatment. Only 17.3% (68) respondents held a contrasting view that malaria was best treated by self-medication.

All 392 participants reported malaria episode in the four weeks prior to the study. Of the 392, 56.6% (222) first treatment action was a visit to the health facility, and the remaining 43.4% (170) used non-prescribed medicines. Of those who sought treatment from the health facility, 62.2% (138) was seen by health professionals. For the respondents who used non-prescribed medicines, 57.6% (98) were adults and 42.4% (72) were children. One respondent reported seeking treatment from the traditional healer. Six-four per cent (250) perceived the malarial symptoms they experienced as very severe. Sixty-two per cent (241) of participants started their treatment within 24 hours of experiencing symptoms while the remaining 38.5% (151) treated after 24 hours of the onset of symptoms.

Non-prescribe drugs used in treating the episode of malaria included anti-malarials 16.8% (66). For the respondents who took non-prescribed anti-malarials, 43.9% (29) took ACT and 56.1% (39) took non-ACT, such as chloroquine, artemether, amodiaquine, quinine, and sulphadoxine-pyrimethamine. Other drugs used in treating episodes of malaria included paracetamol 24% (96) and herbal medicines 14% (55), with about 1.8% (seven) taking antibiotics (Table 1).

**Table 1:** Source and type of treatment for malaria among respondents (n = 392)

**Categories**	**Adults N = 194**	**Child’s caregiver N = 198**	**Overall N = 392**
	**n (%) (95% CI)**	**n (%) (95% CI)**	**n (%) (95% CI)**
**Source**	96 (42.2) (42.2 – 56.7)	126 (56.7) (56.5 – 70.3)	222 (56.6) (51.6 – 61.6)
Health facility for treatment			
Non-prescribed treatment	98 (57.6) (43.3 – 57.8)	72 (42.4) (29.7 – 43.5)	170 (43.4) (38.4 – 48.4)
Traditional healer	1 (0.5) (0.0 – 2.8)	-	1 (0.3) (0.0 – 1.6)
**Drug type**	4 (2.1) (0.6 – 5.2)	3 (1.5) (0.3 – 4.4)	7 (1.8) (0.8 – 3.8)
Took antibiotic			
Took anti-malarial	46 (23.7) (17.9 – 30.3)	20 (10.1) (6.3 – 15.2)	66 (16.8) (11.3 – 21.0)
Paracetamol	45 (23.2) (17.5 – 29.8)	51 (25.8) (6.7 – 15.8)	96 (25.8) (20.4 – 29.1)
Herbs	38 (19.6) (14.2 – 25.9)	17 (8.6) (5.1 – 13.4)	55 (14) (10.8 – 18.0)

Most, 62.3% (106), of those using non-prescribed drugs for treating their malaria obtained them from chemical and pharmacy shops. About 13.5% (23) of respondents relied on leftover drugs from previous malaria episodes, whilst the remaining 24.2% (42) obtained their drugs from neighbours, friends and drug peddlers.

It was established that people who were above five years of age were more likely to use non-prescribed anti-malarial drugs than those aged below five years. Furthermore, respondents influenced by other people to practice self-medication were more likely to use non-prescribed anti-malarial drugs than those who were not influenced [p = 0.001]. The results also suggest that respondents who perceived their malaria symptoms to be mild were more likely to use non-prescribed anti-malarials than those who perceived the symptoms to be severe [p = 0.009]. Respondents who started treatment after 24 hours of onset of symptoms were more likely to use non-prescribed anti-malarials than those who started treatment within 24 hours of onset of symptoms. Respondents who knew the symptoms of malaria were less likely to use non-prescribed anti-malarial drugs than those who did not have such knowledge. Similarly, respondents who knew the cause of malaria were less likely to use non-prescribed anti-malarials than those who did not. Respondents whose main source of treatment for malaria was a health facility were less likely to use non-prescribed anti-malarials (Table 2).

**Table 2:** The use of non-prescribed anti-malarial drugs and respondent attributes

**Attributes**	**Used non-prescribed anti-malarial drug n (%)**		**Chi-square**	**P-value**
	**Yes**	**No**		
*Knows malaria symptoms*				
Yes	60 (15.8)	320 (84.2)	9.72	0.002
No	6 (50)	6 (50)		
*Knows cause of malaria*				
Yes	42 (14.3)	252 (84.7)	5.47	0.019
No	24 (24.5)	74 (75.5)		
*Knows source of malaria treatment*				
Yes	48 (14.4)	285 (85.6)	9.3	0.002
No	18 (30.5)	41 (69.5)		
*Age ≤ 5 years*				
Yes	9 (7)	120 (93)	13.35	0.001
No	57 (21.7)	85 (67.5)		
*Peer/relative influence*				
Yes	25 (56.8)	120 (93)	13.35	<0.001
No	41 (32.5)	85 (67.5)		
*Perception of severity*				
Yes	33 (13.1)	218 (87.6)	6.78	0.009
No	33 (23.4)	108 (76.6)		
*sought treatment within 24 hours*				
Yes	30 (12.4)	211 (87.6)	8.61	<0.001
No	36 (23.8)	115 (76.2)		
*Age ≤ 5 years*				
Yes	9 (7)	120 (93)	13.35	<0.001
No	57 (21.7)	85 (67.5)		

The multiple logistic regression analysis revealed that a predictor of use of non-prescribed anti-malarials among respondents was the influence of other people already practicing self-medication. Respondents who were not influenced by other people to practice self-medication were less likely to use non-prescribed anti-malarials [OR: 0.46 (95% CI 0.22-0.98)] (Table 2).

Among the adult respondents, the use of non-prescribed anti-malarial drugs showed significant correlations using Chi-square test, with factors similar to that of all respondents with the exception of age and influence by other persons on adult respondents to use non-prescribed anti-malarial. The predictors of use of non-prescribed anti-malaria drugs among adult respondents from multiple logistic regression analysis were: adult respondents’ knowledge of treatment of malaria and time to start of treatment. Adult respondents whose main source of seeking treatment for malaria was the health facility were less likely to use non-prescribed anti-malarial drugs than those who did not seek care in facilities (OR: 0.38; 95% CI 0.16-0.89). Adult respondents who started treatment after 24 hours of onset of symptoms were more likely to use non-prescribed anti-malarial drugs than those who started treatment within 24 hours of onset of symptoms (OR: 2.1 95% CI 1.01-4.19) as shown in Table 3.

**Table 3:** Factors affecting use of non-prescribed anti-malarial drugs among adults and children

**Attribute**	**OR**	**95% CI**	**P-value**
*Age ≤ 5 years*			
No	1.00	-	0.287
Yes	1.79	0.613 – 5.22	
*Influenced by others persons’ treatment decision*			
No	1.00	-	0.048
Yes	2.14	1.01 – 4.53	
*Knows cause of malaria*			
No	1.00	-	0.479
Yes	1.31	0.62 – 2.75	
*Knows symptoms of malaria*			
No	1.00	-	0.077
Yes	0.21	0.04 – 1.19	
*Knows source of malaria treatment*			
No	1.00	-	0.139
Yes	0.58	0.28 – 1.20	
*Perceived severity of malaria symptoms*			
Mild	1.00	-	0.731
Severe	0.88	0.44 – 1.79	
Adult			
Child	1.00	-	0.434
	0.71	0.30 – 1.68	

The predictor of use of non-prescribed anti-malarial drugs among children was affected by influence on primary caregivers by other persons to use non-prescribe anti-malarial drugs. Primary caregivers who were influenced by other people were more likely to use non-prescribed anti-malarial drugs to treat their malaria than those who were not influenced (odds ratio: 4.44; 95% CI 1.22,16.10) (Tables 4 and 5).

**Table 4:** Predictors of use of non-prescribed anti-malarial drugs among adult respondents

**Attribute**	**OR**	**95% CI**	**P-value**
*Knows symptoms of malaria*			
No	1.00	-	0.079
Yes	0.25	0.05 – 1.18	
*Knows cause of malaria*			
No	1.00	-	0.208
Yes	0.60	0.27 – 1.33	
*Knows source of malaria treatment*			
No	1.00	-	0.026
Yes	0.38	0.16 – 0.89	
*Started treatment >24 hours of symptoms*			
No	1.00	-	0.046
Yes	2.06	1.01 – 4.19	

**Table 5:** Predictors of use of non-prescribed anti-malaria drugs among children

**Variable**	**OR**	**95% CI**	**P-value**
*Age (Years)*			
≤4	1.00	-	0.222
>4	1.99	0.66 – 6.00	
*Influence by other persons on respondents’ treatment decision*		-	
No	1.00	1.22 – 16.10	0.023
Yes	4.44		
*Perceived severity of malaria symptoms experienced by respondents*			
Mild	1.00	0.20 – 1.82	0.368
Severe	0.60		

## Discussion

The proportion of non-prescribed anti-malarial use among the respondents is reported to be 16.8%. Whereas this finding is consistent with the use of non-prescribed anti-malarials in other settings [1] it is different from a study conducted in the Kassena Nankana District of Upper East Region of Ghana, which reported that 42% of primary caregivers used non-prescribed anti-malarial drugs [3]. The relatively low proportion of non-prescribed anti-malarial drug use in this study is attributable to most respondents having good knowledge of the signs, symptoms and causes of malaria. Coupled with this are low reports on other people’s influence on treatment. Children aged below five years receive health facility treatment when sick. This could be attributable to high number of the inhabitants who have subscribed to the National Health Insurance Scheme and thus are able to access appropriate care from formal health facilities [15].

Although the national policy for the treatment of malaria changed from monotherapy to combination therapy there is still the use of monotherapy drugs [16,17]. This study reveals that respondents who took non-prescribed anti-malarials, a higher proportion 56.1% took non-ACT, such as chloroquine, artemether, amodiaquine, quinine and sulphadoxine-pyrimethamine. This situation can be attributed to the lack of knowledge of chemical sellers about the effective treatment for malaria and are dispensing both ACT and non-ACT anti-malarial drugs to clients to treat malaria [4,6,17,18]. The study also found that children under five years of age were more likely to receive non-prescribed anti-malarials if others influenced their carer’s decision. This finding is a bit alarming as those who are most vunerable to malaria are given non-prescribed anti-malarials and which are most likely to be monotherapy drugs. Most monotherapy drugs are known to have developed resistance to the malaria parasite [8,9,19,20] and their continuous treatment of malaria among under fives could have serious consequences for malaria control efforts.

The study shows remarkable numbers of people treating malaria within 24 hours of onset of symptoms, with many respondents visiting health facilities for treatment. This can be associated with knowing the cause and source of treatment and prevention of malaria. Earlier studies in the region [21,22] have shown low knowledge about cause, treatment and prevention and its associations with high self-medication and low and delayed use of the health facility [3,7,23].

This study points to the use of non-prescribed non-ACT drugs whose effectiveness no longer guarantees cure for malaria [24,25]. The current malaria treatment policy in Ghana and many endemic countries discourages monotherapy and recommends ACT because of its effectiveness in curing malaria. This current research points to the fact that continuous monotherapy to treat malaria could risk the development of resistant to malaria parasites in Africa, as is emerging in Asia [26-29].

## Conclusion

The use of non-prescribed anti-malarial drugs among patients is influenced by respondents’ knowledge, peer influence and perceived severity of symptoms. The high use of non-prescribed anti-malarial drugs in the municipality most of which were monotherapy has implications for the current malaria treatment policy. Although ACTs are very effective in treating malaria, their continuous use without prescription can lead to people taking lower dosage, which can speed up the development of resistance and treatment failure. Increased public education about recommended malaria treatment and increased education of drug sellers about malaria treatment policy can help control the inappropriate use of non-prescribed anti-malarial drugs.

## Competing interests

The authors declare that they have no competing interests.

## Authors’ contributions

AS, PA and PA-B conceived the research idea and were involved in developing the proposal, design and data collection, writing and review of manuscript. MA provided statistical support for data analysis. PW and RA-A undertook a substantial review of the paper. All authors read and approved the final manuscript.
